# Genome-wide association study of nevirapine hypersensitivity in a malawian HIV-infected population

**DOI:** 10.1186/2045-7022-4-S3-P125

**Published:** 2014-07-18

**Authors:** Daniel Carr, Stephane Bourgeois, Mas Chaponda, Elena Cornejo Castro, Panos Deloukas, Munir Pirmohamed

**Affiliations:** 1 University of Liverpool, Molecular and Clinical Pharmacology, UK; 2 Queen Mary University London, William Harvey Research Institute, UK

## 

The non-nucleoside reverse transcriptase inhibitor nevirapine is used in the treatment of HIV in many developing countries. Its use is associated with occurrence of hypersensitivity in 6-10% of patients. This hypersensitivity can manifest as a number of phenotypes which include the severe skin blistering reactions Stevens Johnson syndrome (SJS) and toxic epidermal necrolysis (TEN). The aim was to undertake a genome wide association study (GWAS) in order to identify genetic variants associated with predisposition to nevirapine-induced hypersensitivity. A total of 333 nevirapine-exposed (151 hypersensitive and 182 tolerant) , HIV-infected Malawian adults were genotyped for 826,551 genotyped SNPs using the Illumina HumanOmni1-Quad_v1 chip. A replication cohort of 62 hypersensitive and 59 tolerant patients from Malawi and Uganda was genotyped for 40 SNPs statistically significantly associated with a hypersensitive phenotype in the main cohort (p<5x10-5) using the Sequenom iPLEX platform or TaqMan allelic discrimination. Logistic regression analysis identified 40 statistically significant SNP signals associated with a nevirapine hypersensitivity phenotype. Only 1 SNP association signal (in the HLA-C locus, associated with SJS/TEN) was statistically significant in both our main discovery (=1.48x10-6) and enriched replication cohort (38 cases and 59 controls) (p=9.6x10-5). Meta-analysis determined the odds ratio as 5.17 (p=2.61x10-10). Data suggest this SNP to be a strong proxy for HLA-C*04:01 carriage (96% co-occurrence). We have confirmed that, in a sub-Saharan African population, HLA-C*04:01 carriage confers a significant risk for nevirapine-induced SJS_TEN though not to less severe hypersensitivity phenotypes. No other significant high penetrance genetic risk factors were identified.

